# Moderate-Intensity and High-Intensity Interval Exercise Training Offer Equal Cardioprotection, with Different Mechanisms, during the Development of Type 2 Diabetes in Rats

**DOI:** 10.3390/nu16030431

**Published:** 2024-01-31

**Authors:** Sarah D’Haese, Lisa Claes, Iris de Laat, Sven Van Campenhout, Dorien Deluyker, Ellen Heeren, Sibren Haesen, Ivo Lambrichts, Kristiaan Wouters, Casper G. Schalkwijk, Dominique Hansen, BO Eijnde, Virginie Bito

**Affiliations:** 1 UHasselt, Cardio & Organ Systems (COST), Biomedical Research Institute, Agoralaan, 3590 Diepenbeek, Belgium; sarah.dhaese@uhasselt.be (S.D.); dorien.deluyker@uhasselt.be (D.D.); ellen.heeren@uhasselt.be (E.H.); sibren.haesen@uhasselt.be (S.H.); ivo.lambrichts@uhasselt.be (I.L.); 2 Department of Internal Medicine, CARIM School for Cardiovascular Diseases, Maastricht University Medical Centre, Universiteitssingel 50, 6229 ER Maastricht, The Netherlands; kristiaan.wouters@maastrichtuniversity.nl (K.W.); c.schalkwijk@maastrichtuniversity.nl (C.G.S.); 3 UHasselt, Faculty of Rehabilitation Sciences, REVAL Rehabilitation Research Centre, Agoralaan, 3590 Diepenbeek, Belgium; dominique.hansen@uhasselt.be; 4 Department of Cardiology, Heart Centre Hasselt, Jessa Hospital, Stadsomvaart 11, 3500 Hasselt, Belgium; 5 SMRc-Sports Medicine Research Center, BIOMED-Biomedical Research Institute, Faculty of Medicine & Life Sciences, Hasselt University, 3500 Diepenbeek, Belgium; bert.opteijnde@uhasselt.be; 6 Division of Sport Science, Stellenbosch University, Stellenbosch 7602, South Africa

**Keywords:** type 2 diabetes, diabetic cardiomyopathy, western diet, exercise training, cardioprotection

## Abstract

Endurance exercise training is a promising cardioprotective strategy in type 2 diabetes mellitus (T2DM), but the impact of its intensity is not clear. We aimed to investigate whether and how isocaloric moderate-intensity exercise training (MIT) and high-intensity interval exercise training (HIIT) could prevent the adverse cardiac remodeling and dysfunction that develop T2DM in rats. Male rats received a Western diet (WD) to induce T2DM and underwent a sedentary lifestyle (*n* = 7), MIT (*n* = 7) or HIIT (*n* = 8). Insulin resistance was defined as the HOMA-IR value. Cardiac function was assessed with left ventricular (LV) echocardiography and invasive hemodynamics. A qPCR and histology of LV tissue unraveled underlying mechanisms. We found that MIT and HIIT halted T2DM development compared to in sedentary WD rats (*p* < 0.05). Both interventions prevented increases in LV end-systolic pressure, wall thickness and interstitial collagen content (*p* < 0.05). In LV tissue, HIIT tended to upregulate the gene expression of an ROS-generating enzyme (NOX4), while both modalities increased proinflammatory macrophage markers and cytokines (CD86, TNF-α, IL-1β; *p* < 0.05). HIIT promoted antioxidant and dicarbonyl defense systems (SOD2, glyoxalase 1; *p* < 0.05) whereas MIT elevated anti-inflammatory macrophage marker expression (CD206, CD163; *p* < 0.01). We conclude that both MIT and HIIT limit WD-induced T2DM with diastolic dysfunction and pathological LV hypertrophy, possibly using different adaptive mechanisms.

## 1. Introduction

The global burden of type 2 diabetes mellitus (T2DM) keeps rising persistently, with approximately 783 million adults living with the condition in 2045 [1]. People with T2DM have a two- to fourfold increased risk of developing heart failure compared to those without the disease [2]. The pathophysiology of diabetic cardiomyopathy, a concept covering all diabetes-induced changes in myocardial structure and function in the absence of coronary artery disease and valvular or congenital disorders, develops in two stages [3]. Initially, the presence of insulin resistance, hyperglycemia, glycated proteins or so-called advanced glycation end products (AGEs) stimulates the formation of reactive oxygen species (ROS) and inflammation in the heart [4]. Excessive oxidative stress and immune cell infiltration in the myocardial interstitial space lead to the activation of cardiac fibroblasts, collagen deposition and cardiomyocyte hypertrophy [5]. T2DM patients therefore present diastolic dysfunction, which can develop into heart failure with preserved ejection fraction (EF) (HFpEF, EF ≥ 50%) [6]. Prolonged exposure to myocardial ROS, AGEs and hypoxia, as a consequence of vascular dysfunction, can induce additional interstitial fibrosis and cardiomyocyte damage [4]. These structural alterations might cause reductions in systolic function, characterized by LV dilation and contractile impairment, and might lead to heart failure with reduced ejection fraction (HFrEF, EF ≤ 40%) [6].

Treatment of diabetic individuals with HFpEF or HFrEF currently involves multiple pharmacological approaches with and without device therapies [7]. Additional glucose-lowering agents and compounds that tackle other comorbidities can be considered as well [8]. Although the pharmacological guidelines for heart failure treatment are broad and well-determined, no specific compound to cure both cardiac and metabolic complications in T2DM is currently available. In this regard, the European Society for Cardiology and the American Heart Association have addressed the need to introduce lifestyle changes as basic measures to treat and prevent T2DM with comorbid cardiovascular diseases (CVDs) [7,9]. Endurance exercise training is a promising and cost-effective approach to improving cardiovascular risk factors and metabolic control in diabetic patients with CVDs [10]. Currently, at least 150 min of moderate- or 75 min of vigorous-intensity activity per week are advised to reduce CVD risk [7,9]. However, the most optimal exercise intensity for the management of T2DM in terms of cardioprotection remains unclear. Moderate-intensity exercise training (MIT), which generally occurs continuously at 40–69% of VO_2_max, is considered the gold standard for exercise training prescriptions in T2DM [8]. Regarding cardiac function, a prospective randomized controlled trial has reported that 12-week supervised aerobic exercise training improved diastolic function in asymptomatic adults with T2DM [11]. In the last decade, whether high-intensity interval exercise training (HIIT), characterized by brief bouts of near-maximal effort (70–95% of VO_2_max) alternating with short recovery periods at low intensity, outperforms MIT in cardiovascular benefits has been profoundly discussed [8]. Hollekim et al. have demonstrated that both MIT and HIIT induce diastolic functional improvements at rest in T2DM individuals; however, HIIT seems to be more beneficial than MIT [12]. Conversely, others have found no significant impact of MIT or HIIT on cardiac function in T2DM patients [13]. Individuals with T2DM who have performed HIIT have also had better cardiac autonomic responses, which are associated with cardiovascular events, when compared with moderate-intensity-trained participants [14].

The underlying mechanisms responsible for cardiac improvements after endurance exercise training, predominantly MIT, have been investigated in genetically, chemically and high-fat diet (HFD)-induced T2DM animal models and reviewed elsewhere [15,16]. We recently demonstrated that both MIT and HIIT reverse adverse cardiac remodeling and cardiac dysfunction by lowering myocardial fibrosis and improving mitochondrial oxidative capacity in rats with high-sugar- or so-called Western-diet (WD)-induced T2DM [17]. Up to now, experimental studies have mainly explored the therapeutic effect of endurance exercise training, describing its impact after T2DM diagnosis, on the cardiac phenotype. Nevertheless, the rapid rise in disease prevalence highlights that the prevention of T2DM and associated CVDs should be a key component of public health strategies in the future [18]. Whether endurance exercise training at different intensities can offer cardioprotection, thereby potentially benefitting the diabetic individual at risk, is currently unknown. In our study, we investigated whether MIT and HIIT can prevent adverse cardiac remodeling and dysfunction during the development of T2DM in rats and the underlying mechanisms involved.

## 2. Methods

### 2.1. Animal Experiments and Study Design

All animal experiments were performed in accordance with the guidelines of EU directive 2010/63/EU for animals used for scientific purposes and were approved by the Local Ethical Committee at Hasselt University for Animal Experimentation (Diepenbeek, Belgium; matrix ID202102). All animals were housed in a temperature-controlled environment (21 °C) with an inverse day–night rhythm (12 h light/dark cycle). The animals had access to drinking water and food ad libitum. In total, 27 male Sprague Dawley rats (Janvier Labs, Le Genest-Saint-Isle, France) were used in this study.

**Figure 1:**
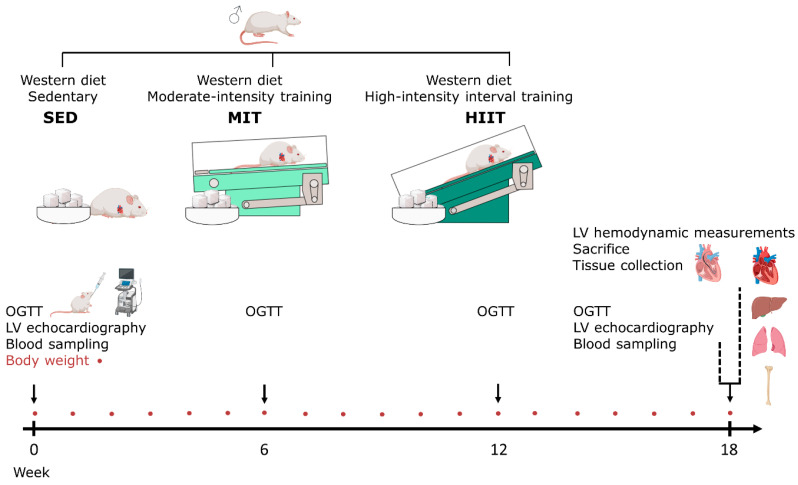
Experimental design of this study. Male Sprague Dawley rats received a Western diet and were randomly assigned to SED (*n* = 7), MIT (*n* = 7) or HIIT (*n* = 8) for 18 weeks in parallel. Body weight, indicated by red circles, was measured weekly. OGTTs were performed at baseline, week 6, week 12 and week 18. LV echocardiography, together with blood sampling, was performed at baseline and week 18. Invasive hemodynamic LV measurements were performed at sacrifice. The heart, liver, lungs and tibia were isolated. HIIT: high-intensity interval training. LV: left ventricular. MIT: moderate-intensity training. OGTT: oral glucose tolerance test. SED: sedentary lifestyle.

The rats, 7 weeks old, received a high-sugar diet or a so-called WD (69% kcal carbohydrates, of which 48% kcal of sugars were from sweetened condensed milk and added sucrose; 16% kcal fat; 15% kcal proteins) daily to induce T2DM with adverse cardiac remodeling and dysfunction, as has been described previously [19,20] (Figure 1). At the onset of the diet, the animals were subjected to a sedentary lifestyle (SED, *n* = 7) or to moderate-intensity training (MIT, *n* = 7) or high-intensity interval training (HIIT, *n* = 8) on a treadmill for 18 weeks. Body weight was measured every week. At baseline, week 6, week 12 and week 18, oral glucose tolerance tests (OGTTs) were conducted. At baseline and week 18, LV echocardiographic measurements and blood sampling were performed. Just before sacrifice, invasive LV hemodynamic measurements were performed. For all procedures, the animals were anaesthetized with 2% isoflurane supplemented with oxygen. At the end of this experiment, the rats were injected intraperitoneally (i.p.) with heparin (1000 u/kg) and sacrificed via injection i.p. with an overdose of sodium pentobarbital (200 mg/kg; Dolethal, Vetoquinol, Aartselaar, Belgium). The hearts, livers and lungs were removed and weighed. Transversal sections at the midventricular level of the heart were cut and fixed in 4% paraformaldehyde overnight. Thereafter, the cardiac sections were transferred to 70% ethanol, followed by paraffin embedding, to perform histological and immunohistochemical staining. Residual LV tissue was crushed into fine powder, subsequently frozen in liquid nitrogen and stored at −80 °C for real-time quantitative polymerase chain reaction (RT-qPCR) evaluation. A hind leg per rat was isolated to measure tibia bone length, which was applied as a normalization factor for organ weights.

### 2.2. Exercise Training Protocol

Running on a treadmill (Expendable Treadmill Model 805; IITC Life Science, Woodland Hills, CA, USA) was conducted for 18 weeks (5 days/week), analogous to a training protocol of Verboven et al. [21]. Prior to the start of exercise training, the rats were acclimatized to the treadmill for two weeks with gradually increased speed and duration of running (3 days/week, 5° inclination), In short, MIT involved continuous moderate-intensity running for 1 h/day (18 m/min, 5° inclination). HIIT consisted of 10 bouts of 2 min high-intensity running (18 m/min, 30° inclination) alternated with 1 min of low-intensity running (12 m/min at 30° inclination). Electrical shock grids at the ends of the treadmill lanes provided mild shocks to stimulate continuous running. Blood lactate levels were measured immediately after exercise training with an Analox GM7 Micro-Stat Analyzer (Analis, Namur, Belgium) to evaluate the exercise intensity. A blood lactate level of >4 mmol/L was acknowledged as an indicator of high-intensity exercise training. The training modalities were modified to achieve an equal energy expenditure by calculating the net caloric cost (kcal/min) using two formulas [21]:

VO_2_ max = S * 0.2 + (S * G) * 0.9 (S = speed (m/min), G = inclination)

Net caloric cost (kcal/min) = VO_2_ max * 3.5 * body mass (kg)/200

### 2.3. Conventional Echocardiographic Measurements

Transthoracic LV echocardiography was conducted in all rats with a 21 MHz MX250 transducer and a Vevo^®^ 3100 high-resolution imaging system (FUJIFILM VisualSonics, Inc., Amsterdam, The Netherlands), as has been described by Evens et al. [22]. During scanning, physiological parameters, including heart rate (HR) and ECG signals, were monitored non-invasively. From parasternal short-axis views in M-mode, the left ventricular posterior wall thickness in the diastole and systole (PWTd and PWTs) and the anterior wall thickness in the diastole and systole (AWTd and AWTs) were determined, for which the measurements of three heartbeats were averaged. End-systolic and end-diastolic volumes (ESVs and EDVs) and cardiac output (CO) were obtained from B-mode parasternal long-axis views. CO was normalized to the body surface area (BSA) and expressed as cardiac indexes. Apical four-chamber views in B-mode were used to assess mitral flow profiles. Pulsed-wave Doppler mode was used to obtain the ratio of peak mitral flow velocity in early versus late filling (E/A). In addition, peak septal mitral annulus velocity in early filling phase (E′) was assessed with tissue Doppler. For pulsed-wave and tissue Doppler, the measurements from two cycles were averaged. The E/E′ ratio was calculated. Echocardiographic images were analyzed using Vevo^®^ LAB software (Vevo^®^ LAB software, version 5.6.1; FUJIFILM VisualSonics, Inc.). All echocardiographic images were analyzed by two blinded investigators.

### 2.4. Hemodynamic Measurements

Invasive pressure LV measurements were performed at sacrifice, as has been described by Verboven et al. [19]. In short, hemodynamic measurements were performed with a precalibrated SPR-320 MikroTip high-fidelity pressure catheter (Millar Inc., The Hague, The Netherlands), which was inserted into the LV area via the right carotid artery. The catheter was linked to a quad-bridge amplifier and a PowerLab 26T module (AD Instruments, Oxford, UK) to load data into LabChart software (LabChart v7.3.7 software, AD Instruments). Hemodynamic parameters, including end-diastolic pressure (EDP), end-systolic pressure (ESP), diastolic and systolic duration, the maximum and minimum peak time derivatives (dP/dt_max_ and dP/dt_min_, respectively), the systolic pressure–time index (SPTI) and the time constant for isovolumetric relaxation (Tau), were measured.

### 2.5. Oral Glucose Tolerance Test and Insulin Resistance Assessment

Glucose tolerance was assessed with a 1 h OGTT each at baseline, week 6, week 12 and week 18 after the onset of the diet. After a 16 h overnight fast, the rats received glucose (2 g/kg) by oral gavage. Before glucose administration, blood glucose concentration was measured in blood collected from the capillary tail with an Analox GM7 Analyzer (Analis SA, Namur, Belgium). Capillary tail blood collection was repeated 15, 30 and 60 min after glucose administration. The glucose response was expressed as the total area under the curve (AUC). At baseline and after 60 min, plasma insulin concentration was measured using an electrochemiluminescence assay (Mouse/Rat Insulin Kit, K152BZC; Meso Scale, Gaithersburg, MD, USA). To assess insulin resistance, the homeostasis model assessment of insulin resistance (HOMA-IR) was calculated from the fasting glucose and insulin concentrations using the following formula:

HOMA−IR=(fasting insulin [µIU/mL] ∗ fasting glucose [mmol/L])/22.5

### 2.6. Histology

Paraffin-embedded transverse sections of LV tissue (7 μm thickness) were obtained at the midventricular level of the heart. Sections were stained with use of the Sirius Red/Fast Green Collagen Staining kit (9046; Chondrex Inc., Woodville, TX, USA), following the manufacturer’s instructions. After staining, the sections were dehydrated in increased ethanol concentrations and mounted with a DPX mounting medium. Images were captured with a Leica MC170 camera connected to a Leica DM2000 LED microscope (Leica Microsystems, Diegem, Belgium). Areas of collagen deposition, visualized with red-to-purple staining, was quantified in the LV areas of the rats in four randomly chosen and blinded images per section using the color deconvolution plugin in Fiji v1.53c software [23]. The area of collagen deposition was normalized to the total cardiac area and expressed as a percentage of collagen deposition. Perivascular fibrosis was determined by normalizing the fibrotic area, determined with the color threshold tool in the Fiji v1.53c software, to the luminal area, quantified with the assumption of an elliptical cross-section and expressed as a percentage of collagen deposition. At least six blood vessels located in the LV area were used to determine perivascular fibrosis. Histological analyses were performed by two researchers independently.

### 2.7. Immunohistochemistry

Paraffin-embedded transverse sections of LV tissue (7 µm thickness) were stained for cluster of differentiation 68 (CD68), advanced glycation end products (AGEs) and lysyl oxidase (LOX). For CD68 and AGE staining, deparaffinized sections underwent heat-mediated antigen retrieval using a citrate buffer (pH = 6). Endogenous peroxidase was blocked with 30% hydrogen peroxide diluted at 1:100 in PBS for 20 min at room temperature (RT). The sections were permeabilized with 0.05% Triton X-100 (Merck Life Science BV, Overijse, Belgium) for 20 min at RT and blocked with a serum-free protein block (X0909; Dako, Agilent Technologies, Diegem, Belgium) for 20 min at RT to minimize background staining. Tissue sections were incubated with a primary antibody against CD68 (1:100, 1 h at RT, mouse monoclonal MCA341R; Bio-Rad, Temse, Belgium), AGEs (1:250, 1 h at RT, rabbit polyclonal ab23722; Abcam, Cambridge, UK) or LOX (1:200, overnight at 4 °C, rabbit polyclonal ab31238; Abcam) diluted in PBS. EnVision™ with Dual-Link System horseradish peroxidase (K4061, 30 min at RT, antirabbit/antimouse; Dako) was used for CD68 and AGEs. A horseradish peroxidase-conjugated secondary antibody (1:400, 30 min at RT, P0448, goat polyclonal; Dako) was used for LOX. Negative controls, incubated without a primary antibody, were included in each staining. All sections were incubated with 3,3′-diaminobenzidine (Dako) and were counterstained with hematoxylin to stain the nuclei. The sections were mounted using a DPX mounting medium. Images were obtained using a Leica MC170 camera that was connected to a Leica DM2000 LED microscope (Leica Microsystems). The AGE and LOX deposition were quantified in the LV area in four randomly chosen and blinded sections per section with use of the color deconvolution plugin in the Fiji v1.53c software [23]. This area was normalized to the total cardiac area and expressed as a percentage. CD68 staining was assessed semi-quantitatively with the following scores: absent or limited staining (0), minor staining (1), moderate staining (2), high staining with the presence of small aggregates (3) and high staining with the presence of large aggregates (4). Analyses were performed blinded for group allocation by two independent researchers.

### 2.8. RT-qPCR

The total RNA was isolated from snap-frozen LV tissue (30 mg) using an RNeasy Fibrous Tissue Kit (Qiagen Benelux B. V., Antwerp, Belgium), following the manufacturer’s instructions. The concentration and purity of the RNA were assessed using a NanoDrop 2000 spectrophotometer (Isogen Life Science B. V., Utrecht, The Netherlands). cDNA synthesis was performed using a qScript cDNA SuperMix (QuantaBio, VWR International bvba, Leuven, Belgium). Primers were designed in the coding sequence of the mRNA (Integrated DNA Technologies, Leuven, Belgium) (Table S1). The expressions of cluster of differentiation 163 (CD163), cluster of differentiation 206 (CD206), CD68, cluster of differentiation 86 (CD86), glyoxalase 1 (GLO1), interleukin 1 beta (IL-1β), nicotinamide adenine dinucleotide phosphate (NADPH) oxidase 4 (NOX4), the receptor for advanced glycation end products (RAGE), superoxide dismutase 2 (SOD2) and tumor necrosis factor alpha (TNF-α) were studied. RT-qPCR was carried out in a MicroAmp™ Fast Optical 96-well reaction plate with SYBR Green (Thermofisher Scientific, Geel, Belgium) using the QuantStudio 3 PCR system (Thermofisher Scientific). Analysis of gene expression data was performed via the ∆∆CT method following the MIQE guidelines (58). The most stable reference genes for this experiment (ribosomal protein L13a (RPL13a) and hydroxymethylbilane synthase (HMBS)) were determined with geNorm.

### 2.9. Quantification of Circulating Cardiac Injury Biomarkers

After the blood samples were centrifuged at 2× *g* (10 min, 4 °C), plasma was collected and stored at −80 °C. Plasma troponin I (TnI), fatty acid binding protein (FABP) and myosin light chain 3 (Myl3) protein levels were measured using the Cardiac Injury Panel 3 (rat) Kit (K15161C; Meso Scale Discovery (MSD), Rockville, MD, USA), following the manufacturer’s instructions. In brief, standards and plasma samples were added to plates precoated with the primary capture antibodies of interest. Then, electrochemiluminescent MSD SULFO-TAG detection antibodies were added, followed by the addition of MSD Read Buffer T. The plasma TnI, FABP and Myl3 concentrations were determined using electrochemiluminescence (Meso Scale, Gaithersburg, MD, USA). The unknown cardiac injury marker concentrations were calculated using comparison with the standard curves.

### 2.10. Statistical Analysis

Graphpad Prism (Graphpad Software, version 9.3.0; San Diego, CA, USA) was used to conduct statistical analysis. Power analysis was conducted via G*Power (G*Power software, version 3.1.9.4; Düsseldorf, Germany) to determine the sample size. The ROUT test method, with a maximum desired false discovery rate of 1%, was used to detect outlier values. Outliers were excluded for further analysis. The normal distribution of the data was evaluated with use of the Shapiro–Wilk test. Normally distributed data were subsequently analyzed with a parametric one-way ANOVA followed by Tukey’s multiple comparison test. Non-normally distributed data were subjected to a non-parametric Kruskal–Wallis test followed by Dunn’s multiple comparison test. Data measured at multiple time points were compared using a repeated-measures two-way ANOVA followed by the Bonferroni post hoc correction. Simple linear regression models were used to determine the relationships between distinct variables. All data are expressed as means ± standard errors of the means (SEMs). The sample size is indicated as ‘n’. *p* < 0.05 was considered statistically significant.

## 3. Results

### 3.1. Exercise Training Prevents Body and Heart Weight Gain

**Figure 2:**
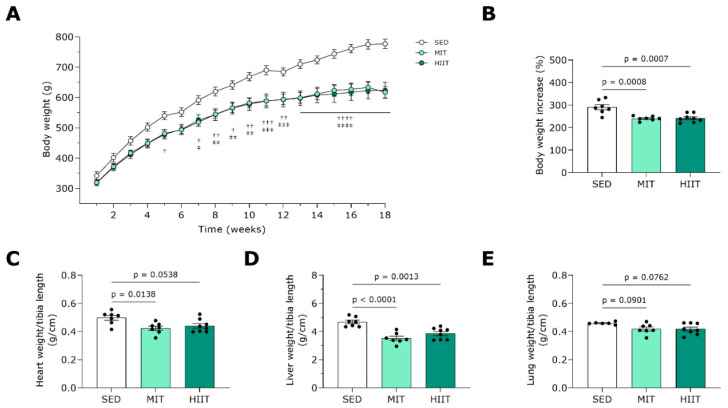
Animal characteristics of Western-diet-fed rats subjected to exercise training. Biometric characteristics of all groups, including SED (*n* = 6–7), MIT (*n* = 7) and HIIT (*n* = 8). (**A**) Progression of body weight over time. (**B**) Body weight at week 18, normalized to body weight at baseline, expressed as a percentage. (**C**) Heart weight to tibia length ratio. (**D**) Liver weight to tibia length ratio. (**E**) Wet lung weight to tibia length ratio. Data represent means ± SEMs. † denotes *p* < 0.05, †† denotes *p* < 0.01, ††† denotes *p* < 0.001 and *p* < 0.0001 denotes †††† SED vs. MIT. ‡ denotes *p* < 0.05, ‡‡ denotes *p* < 0.01, ‡‡‡ denotes *p* < 0.001 and *p* < 0.0001 denotes ‡‡‡‡ SED vs. HIIT.

MIT and HIIT significantly prevented gains in body weight in WD-fed rats (Figure 2A,B). The exercise-trained rats displayed decreases in the heart weight to tibia length ratio (Figure 2C). Furthermore, both exercise training modalities decreased liver weight (Figure 2D) and tended to lower wet lung weight (Figure 2E).

### 3.2. Exercise Training Improves Glucose Tolerance and Insulin Sensitivity

**Figure 3:**
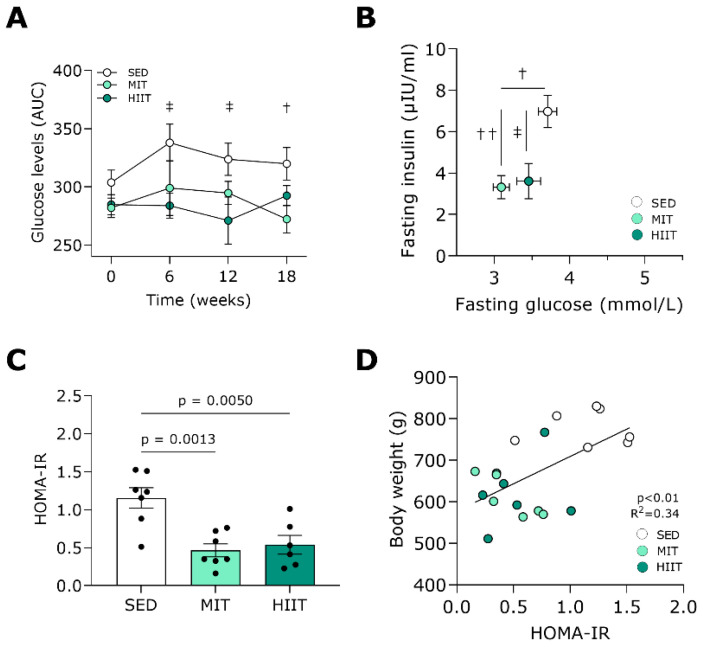
Exercise training improved glucose tolerance and insulin sensitivity in Western-diet-fed rats. (**A**) Glucose levels over time during a 1 h OGTT in plasma from SED (*n* = 7), MIT (*n* = 7) and HIIT (*n* = 8) groups, expressed as AUCs. (**B**) Insulin levels, as a function of glucose levels, measured after overnight fasting during an OGTT in plasma from SED (*n* = 7), MIT (*n* = 7) and HIIT (*n* = 6) groups. (**C**) Calculated HOMA-IR index from SED (*n* = 7), MIT (*n* = 7) and HIIT (*n* = 6) groups. (**D**) Correlation between HOMA-IR values and body weight from SED (*n* = 7), MIT (*n* = 7) and HIIT (*n* = 6) groups. Data represent means ± SEMs. † denotes *p* < 0.05, †† denotes *p* < 0.01 SED vs. MIT and ‡ denotes *p* < 0.05 SED vs. HIIT. AUC: area under the curve. HOMA-IR: homeostasis model assessment of insulin resistance. OGTT: oral glucose tolerance test.

Both exercise training protocols prevented increases in the total glucose levels in the plasma of the rats that received WD over time (Figure 3A). At week 18, the moderate-intensity-trained animals had significantly lower fasting plasma glucose concentrations compared with the SED animals (Figure 3B). In addition, both MIT and HIIT significantly prevented the increases in fasting plasma insulin concentrations seen in the SED rats. In line, the HOMA-IR values were significantly lower with both exercise training modalities (Figure 3C). Furthermore, they positively correlated with body weight (*p* < 0.01, R^2^ = 0.34; Figure 3D).

### 3.3. Exercise Training Ameliorates Diastolic Function and Prevents Adverse LV Cardiac Remodeling

At the end of this study, echocardiography was performed to evaluate LV cardiac function and structure, and the measurements are shown in Table 1. Overall, LV structure and function were improved in the moderate-intensity- and high-intensity interval-trained animals. Both MIT and HIIT lowered the PWTd and PWTs, hallmarks of a hypertrophic LV wall, seen in the SED animals. Representative echocardiographic M-mode images for each group are demonstrated in Figure S1A. Furthermore, the EDV and ESV, indicators of LV chamber dilation, as well as the cardiac index, remained comparable in all groups. Notably, both exercise training modalities significantly decreased radial FS, while only the HIIT tended to reduce EF compared to SED (*p* = 0.08, Table 1). The protein levels of plasma TnI, FABP and Myl3, biomarkers of cardiac injury, were unchanged following exercise training (Figure S1B–D).

Hemodynamic measurements of LV function were performed at sacrifice and are shown in Table 2. Exercise training ameliorated diastolic function, as MIT tended to decrease EDP (*p* = 0.06, Table 2) and both modalities significantly lowered Tau, the time constant for isovolumetric relaxation. Regarding systolic function, MIT and HIIT decreased ESP and SPTI, indicators of hypertension and cardiac workload, respectively. 

### 3.4. Exercise Training Limits LV Fibrosis

**Figure 4:**
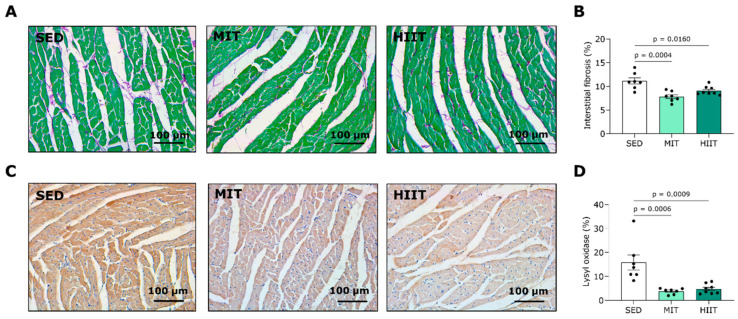
Exercise training prevented LV fibrosis and collagen crosslinking in Western-diet-fed rats. (**A**) Representative pictures of LV tissue stained with Sirius Red/Fast Green. Fibrotic tissue is stained red/purple while cardiac tissue is stained green. (**B**) Quantification of the percentage of interstitial collagen deposition per surface area in LV tissues from SED (*n* = 7), MIT (*n* = 7) and HIIT (*n* = 8) groups. (**C**) Representative pictures of LV tissues stained for lysyl oxidase. (**D**) Quantification of lysyl oxidase staining in LV tissues from SED (*n* = 7), MIT (*n* = 7) and HIIT (*n* = 8) groups. Data represent means ± SEMs. LV: left ventricular.

Representative images of LV sections stained with Sirius Red/Fast Green, visualizing interstitial collagen, are shown in Figure 4A. MIT and HIIT significantly prevented LV interstitial collagen deposition, as seen in the SED animals, aligning with the decreased wall thickness and improved diastolic function observed in the echocardiography and hemodynamic measurements. Accordingly, the alterations in the LV interstitial fibrosis correlated positively with changes in the PWTd (Figure S1E) and Tau (Figure S1F). To evaluate the mechanism responsible for the preventive effect of exercise training on cardiac interstitial fibrosis, the LV tissue was stained for LOX, a protein involved in the crosslinking of collagen fibrils (Figure 4C). The moderate-intensity- and high-intensity interval-trained rats had significantly lower LOX protein expression levels in the LV area compared with the SED animals (Figure 4D). Contrary to the findings on interstitial fibrosis, the MIT and HIIT increased collagen deposition at perivascular sites (Figure S2).

### 3.5. Exercise Training Triggers LV Oxidative Stress and Inflammation but Also Upregulates Protective Mechanisms

**Figure 5:**
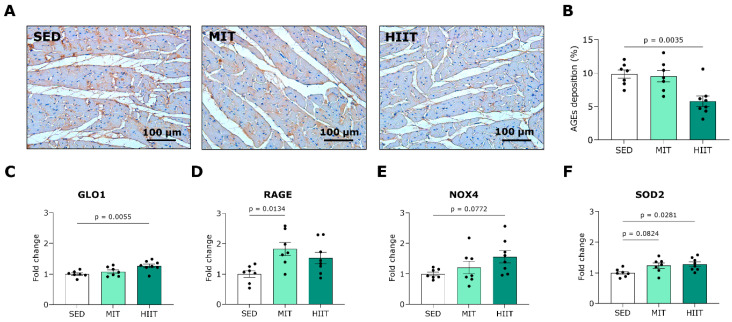
Exercise training decreased advanced glycation end-product content and improved redox balance in the LV areas of Western-diet-fed rats. (**A**) Representative pictures of LV tissues stained for total AGE deposition (brown). (**B**) Quantification of AGE deposition in LV tissues from SED (*n* = 7), MIT (*n* = 7) and HIIT (*n* = 8) groups. (**C**,**D**) Quantification of expression of genes involved in AGE pathways, namely (**C**) GLO1 and (**D**) RAGE, in LV tissues from SED (*n* = 7), MIT (*n* = 7) and HIIT (*n* = 8) groups. (**E**,**F**) Quantification of expression of genes involved in redox balance, namely (**E**) NOX4 and (**F**) SOD2, in LV tissues from SED (*n* = 7), MIT (*n* = 7) and HIIT (*n* = 8) groups. Data represent means ± SEMs. AGEs: advanced glycation end products. GLO1: glyoxalase. LV: left ventricular. NOX4: nicotinamide adenine dinucleotide phosphate oxidase 4. SOD2: superoxide dismutase 2. RAGE: receptor for AGEs.

To further investigate the underlying cardioprotective mechanisms of exercise training, we examined the proteins and genes involved in the cardiac AGE pathways, redox balance and inflammation. Total AGE content was semi-quantitatively analyzed in LV sections via immunohistochemistry, and representative images are provided in Figure 5A. HIIT significantly decreased AGE deposition in LV tissue compared to SED (Figure 5B).

In line, the gene expression of GLO1, an enzyme that inhibits dicarbonyl stress, was upregulated in the high-intensity interval-trained animals compared with the SED animals (Figure 5C). Furthermore, the MIT significantly induced a higher expression of RAGE, the key receptor for AGEs, at the gene level (Figure 5D). Regarding oxidative stress, the HIIT tended to cause an elevated gene expression of ROS-generating enzyme NOX4 (Figure 5E). The rats that underwent MIT and HIIT showed increased expression of antioxidative gene SOD2 compared with the SED rats (Figure 5F).

**Figure 6:**
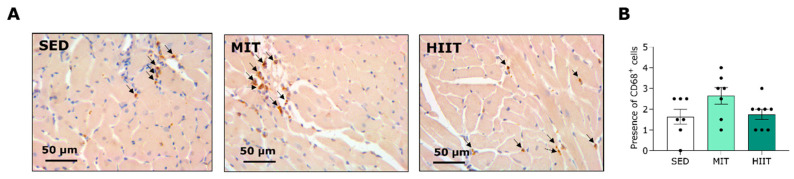

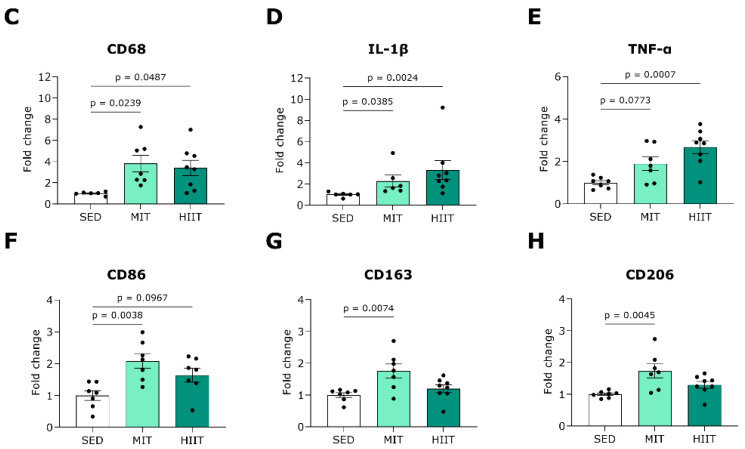
Exercise training induced pro- and anti-inflammatory macrophage infiltration in the LV areas of Western-diet-fed rats. (**A**) Representative pictures of CD68^+^ cells (brown, indicated by black arrows) in LV tissue. (**B**) Semi-quantification of CD68^+^ cells in LV tissues from SED (*n* = 7), MIT (*n* = 7) and HIIT (*n* = 8) groups. Scoring: absent or limited (0), minor (1), moderate (2), high with small aggregates (3) and high with large aggregates (4). (**C**) Quantification of the gene expression of CD68 in LV tissues from SED (*n* = 6), MIT (*n* = 7) and HIIT (*n* = 8) groups. (**D**,**E**) Quantification of the gene expression of proinflammatory cytokines (**D**) IL-1β and (**E**) TNF-α in LV tissues from SED (*n* = 6–7), MIT (*n* = 7) and HIIT (*n* = 8) groups. (**F**–**H**) Quantification of the gene expression of proinflammatory macrophage marker CD86 and anti-inflammatory macrophage markers (**G**) CD163 and (**H**) CD206 in LV tissues from SED (*n* = 7), MIT (*n* = 7) and HIIT (*n* = 7–8) groups. Data represent means ± SEMs. CD163: cluster of differentiation 163. CD206: cluster of differentiation 206. CD68: cluster of differentiation 68. CD86: cluster of differentiation 86. IL-1β: interleukin 1 beta. LV: left ventricular. TNF-α: tumor necrosis factor alpha.

Furthermore, the MIT induced the infiltration of cells positive for CD68, a pan-macrophage marker, in the LV tissues of the WD-fed rats (Figure 6A,B). Both the moderate-intensity- and high-intensity interval-trained rats displayed significantly elevated expression of CD68 at the gene level compared with the SED rats (Figure 6C). Notably, the gene expression of proinflammatory cytokines IL-1β and TNF-α in the LV area was also provoked with exercise training (Figure 6D,E). To evaluate possible differences in macrophage subsets, the expression of macrophage markers at the gene level was measured. The gene expression of CD86, a proinflammatory macrophage marker, was upregulated in the LV tissues of both the moderate-intensity- and high-intensity interval-trained animals compared with the SED rats (Figure 6F). In addition, the MIT induced significantly higher expression of anti-inflammatory macrophage markers CD163 and CD206 in the LV areas of the WD-fed rats.

## 4. Discussion

The current study is the first to have uncovered the cardioprotective impacts of different exercise intensities in the development of T2DM. We have demonstrated that MIT and HIIT equally limit adverse LV remodeling and diastolic dysfunction, possibly using different underlying mechanisms, in a WD-induced rat model of T2DM.

### 4.1. MIT and HIIT as Cardioprotective Strategies in the Development of T2DM

Prospective cohort studies have demonstrated that long-term consumption of sugar-sweetened beverages is positively correlated with incidental T2DM [24] as well as CVD mortality [25]. Preclinically, diets high in both fat and sucrose have been shown to induce T2DM with LV hypertrophy and diastolic dysfunction in mice after 16 weeks and 17 weeks, respectively [26,27]. Following injection with a pancreatic β-cell toxin, rats that were fed a high-sucrose, HFD displayed worse adverse LV remodeling and diastolic dysfunction compared with moderate-sucrose, HFD-fed rats [28]. To mimic this clinical situation, our research group previously developed a rat model of T2DM induced by a high-sucrose diet or the WD, with adverse cardiac remodeling and cardiac dysfunction [19]. In this model, the WD for 18 weeks led to weight gain, insulin resistance and disturbed glucose tolerance, as well as cardiac complications, including LV hypertrophy and increased EDP, while the EF remained preserved. When the WD regime was continued, the rats displayed hypertension, increased ESV and pronounced reduction in EF by 30 weeks [17]. Metabolically, we have now shown that both MIT and HIIT are able to prevent the development of insulin resistance and improved glucose tolerance in WD-fed rats, confirming results in clinical trials on patients at risk for T2DM, as has been reviewed elsewhere [29].

In the heart, physiological eccentric hypertrophy, characterized by a mild increase in LV size and preserved cardiac morphology and function, can develop in response to endurance training [30]. However, we have previously demonstrated that 12-week MIT and HIIT have reversed pathological concentric hypertrophy, characterized by increased PWT, heart weight and cardiac fibrosis, in WD-fed rats [17]. The potential of endurance exercise training to treat pathological concentric hypertrophy has also been confirmed in a randomized controlled trial that showed that high-intensity exercise training reduced LV myocardial stiffening in HFpEF patients with LV septal hypertrophy [31]. Interestingly, Howden et al. demonstrated in a prospective cohort study that endurance exercise training also prevents the increase in cardiac stiffness attributable to sedentary aging in healthy, formerly sedentary participants [32]. Here, we have shown for the first time that both MIT and HIIT significantly prevent increases in PWT and heart weight in rats fed a WD, indicating the protective nature of these exercise training modalities against pathological concentric hypertrophy in developing T2DM. Furthermore, we have shown that MIT and HIIT halt increases in EDP and Tau, both indicators of early diastolic dysfunction, in WD-fed rats. Accordingly, studies of HFD-induced T2DM mice and rats have shown improvements in diastolic hemodynamics, including EDP, Tau and dP/dt_min_, following prolonged MIT and/or HIIT [33,34,35].

Regarding systolic function, several experimental studies have reported increased EF and FS after prolonged MIT and HIIT in HFD-induced T2DM rodent models [36,37,38]. The latter was also confirmed by a clinical study by Cassidy et al., showing that endurance exercise training at high intensity improved both stroke volume and EF in patients suffering from T2DM [39]. Here, we have demonstrated that MIT and HIIT could prevent hypertension but high-intensity interval-trained rats tend to have decreased EF, suggesting worse systolic function with high-intensity exercise training. Nevertheless, a recent clinical trial by De Bosscher et al. has confirmed that mildly reduced EF frequently occurs in asymptomatic endurance athletes (i.e., athlete’s heart) but is not associated with detrimental structural or functional cardiac changes [40]. This is in line with our findings, as the cardiac injury biomarker levels in the plasma of the moderate-intensity- and high-intensity interval-trained rats were unchanged when compared with the sedentary WD-fed rats.

### 4.2. Mechanisms of Exercise-Training-Induced Cardioprotection

Fundamentally, exercise training can be considered a stressor during and after its execution, potentially inducing both beneficial and detrimental health effects [41]. Depending on the exercise volume (i.e., duration and intensity), exercise training might lead to perturbations in redox homeostasis and inflammatory changes, thereby activating adaptive strategies that aim to set a new equilibrium. This study provides new insights into the underlying mechanisms of endurance exercise training-induced cardiac adaptations in rats that have developed T2DM.

In diabetic cardiomyopathy, excessive formation of LV interstitial fibrosis is one of the main disease hallmarks and underlies pathological concentric remodeling [3]. We and others have previously shown that MIT and HIIT have therapeutically decreased interstitial fibrosis content in the LV areas of diet-induced T2DM rats with cardiac dysfunction [17,38,42]. Here, we have validated these findings in preventive settings, as both exercise training modalities reduced LV interstitial fibrosis. Furthermore, the MIT and HIIT both resulted in lower myocardial content of LOX, which favors the crosslinking of the extracellular matrix (ECM) fibers collagen and elastin [43]. In aging hypertensive rats, MIT has also been shown to exert antifibrotic and -crosslinking properties by reducing cardiac interstitial fibrosis and LOX gene expression [44]. Contrary to our findings on LV interstitial fibrosis, we observed that the MIT and HIIT increased fibrosis around the LV intramyocardial blood vessels of the WD-fed rats. Rubies et al. found that HIIT but not MIT promoted aortic stiffening caused by fibrotic remodeling of the tunica media, which was mediated by microRNAs (i.e., miR-212/132 and miR-146b), in healthy rats, confirming the potential of endurance exercise training to induce fibrotic adaptations of the vascular wall [45].

The formation and accumulation of AGEs play an important role in the pathology of T2DM and its cardiac complications [46,47]. Methylglyoxal (MGO), a highly reactive dicarbonyl compound, is the main byproduct of glycolysis and an important precursor of AGEs [48]. In physiological conditions, the majority of MGO becomes detoxified by GLO1, the rate-limiting enzyme of the glyoxalase system. Only a few studies have described how endurance exercise training is related to lower MGO stress and AGE levels in blood and tissues. A cross-sectional study by Maessen et al. has described how lifelong endurance athletes have lower circulating MGO compared with sedentary controls [49]. Preclinically, we have shown that HIIT has decreased plasma AGEs in WD-induced T2DM rats [17]. MIT has also attenuated the accumulation of total AGE content in the myocardia of aging rats [50]. Here, we have found that HIIT reduced the total AGE content, predominantly situated in the ECM, and upregulated the gene expression of GLO1 in the myocardium, potentially indicating HIIT-induced detoxification of MGO-derived AGEs. Future research should identify which specific AGEs, especially in cardiac tissue and plasma, are affected by endurance exercise training via ultra-performance liquid chromatography tandem mass spectrometry [51]. Besides forming ECM crosslinking modifications, AGEs can also bind to their membrane receptor, RAGE, on different cells, thereby initiating intracellular oxidative stress and inflammation states [46]. Here, we have shown that MIT upregulated the LV gene expression of RAGE in WD-fed rats. Al-Robaiy et al. have demonstrated that mice that performed 8 months of voluntary treadmill running had more leukocyte infiltration in their lung tissue, which was not observed in the lungs of RAGE knockout mice, suggesting RAGE as a mediator of tissue inflammation caused by endurance exercise training [52].

NADPH oxidases, especially NOX4, are major sources of ROS in the diabetic heart, and their activity is stimulated by chronic high glucose levels [53]. NOX4 is located at the internal mitochondrial membrane, where it produces hydrogen peroxide and, to a lesser extent, superoxide radicals. There is growing evidence that moderate-to-vigorous intensity endurance exercise training leads to increased ROS production [54]. Endurance athletes have had increased serum malondialdehyde levels, a marker of oxidative stress-induced lipid peroxidation, after daily HIIT workouts for 8 weeks [55]. Accordingly, we observed that 18 weeks of HIIT, and not MIT, tended to increase the gene expression of NOX4 in the LV tissues of the WD-fed rats, suggesting elevated mitochondrial ROS production in the heart. In addition, the exercise-induced hormesis concept describes how ROS production is important to initiating the physiological adaptation of the skeletal muscle to exercise training [54]. In the heart, upregulated NOX4 has been shown to activate nuclear transcription factor erythroid 2-related factor 2, thereby increasing antioxidant SOD2 in cardiomyocytes isolated from healthy mice following short-term MIT [56]. In the current study, we observed significantly increased gene expression of SOD2 in both the moderate-intensity- and high-intensity interval-trained WD-fed animals. In line with our findings, a study of healthy mice has demonstrated upregulated protein expression of NOX4 and SOD2 in the myocardium following stress exercise testing, suggesting an antioxidative feedback response [57]. To clarify the role of exercise-induced oxidative stress in diet-induced diabetic cardiomyopathy models, direct measurements of cardiac ROS should be performed.

In the past decade, interest has grown in the contributing roles of different inflammatory cells, especially macrophages, and their secreted cytokines in the development of CVDs in T2DM [58]. Macrophages are typically classified into proinflammatory (i.e., classically activated) macrophages, which secrete IL-1β and TNF-α, and anti-inflammatory (i.e., alternatively activated) macrophages, although it is becoming increasingly clear that this dichotomous division is an oversimplification of macrophage activation states in vivo [59]. To date, the literature has shown no consensus on the effect of exercise training, especially its intensity and duration, on systemic and cardiac inflammation in metabolic diseases. As such, HIIT has not changed the circulating concentrations of C-reactive protein, IL-1β and TNF-α in older adults at risk for T2DM [60]. In the hearts of mice fed HFDs in the presence or absence of high fructose, the protein levels of IL-1β and/or TNF-α were lower with prolonged MIT [34,61]. Contrary to these findings, we are the first to have observed that both long-term MIT and HIIT have increased the LV gene expression of proinflammatory macrophage markers and cytokines in WD-fed rats. Although this is being tested in the sera of healthy endurance athletes, exhaustive exercise training has also been shown to be associated with increased expression of the proinflammatory cytokines interleukin 6 and 8 [62]. Interestingly, a study by Ostrowki et al. has reported that prolonged exercise training has induced increases in IL-1β and TNF-α serum levels in healthy athletes but that this is counteracted by the release of anti-inflammatory cytokine interleukin 10 [63]. Accordingly, we observed that MIT increased the LV gene expression of anti-inflammatory macrophage markers CD163 and CD206. In the livers of HFD-induced T2DM mice, upregulation of CD163 and CD206 has also been demonstrated following 8 weeks of HIIT [64]. Together, these findings suggest that endurance exercise training can act predominantly as an anti-inflammatory approach in T2DM.

### 4.3. Study Limitations

First, we need to acknowledge that our study was limited to male rats. However, it is important to investigate both sexes, as sex-specific differences exist in the development of T2DM and its cardiovascular complications [65]. Furthermore, cardiac adaptations induced by exercise, concerning intensity and duration, have also been reported to differ between males and females [66]. Since most preclinical studies have focused on male rodents, the impact of exercise training on diabetic cardiomyopathy remains to be determined in female rodents [17,33,34,35,36,37,38,42]. Secondly, this study could not pinpoint one dominant pathway but rather has suggested multiple mechanisms underlying endurance exercise training-induced cardioprotection in T2DM, providing a first indication of their involvement. Research using pathway-specific inhibitors is eagerly anticipated, as this might untangle the causal role of endurance exercise on fibrosis, AGEs, oxidative stress and inflammation in diabetic cardiomyopathy. Based on the findings of the current study, further investigation into the effects of endurance exercise training on cardiomyocyte hypertrophy during the development of T2DM is of special interest. In addition, obtaining insights into the potential beneficial effects of MIT and HIIT on the structure and function of cardiomyocytes isolated from diabetic hearts is needed in the future. Lastly, since former clinical research has mainly focused on the curative role of endurance exercise training in this disease, the clinical implications of the present study, designed in a preventive setup, remain unclear. Prospective studies of different exercise intensities in individuals at risk for T2DM and CVD are therefore warranted in the future.

## 5. Conclusions

This study provides novel insights into the potential of endurance exercise training at moderate and high intensity to prevent the development of WD-induced T2DM with cardiac dysfunction in rats. Regarding metabolic effects, both MIT and HIIT prevented the onset of insulin resistance and ameliorated glucose tolerance over time. In the heart, both exercise interventions prevented the development of diastolic dysfunction and hypertension. Together, our findings demonstrate that both prolonged MIT and HIIT are effective in preventing WD-induced diabetic cardiomyopathy in rats, possibly using different underlying mechanisms, suggesting that both interventions are suitable as cardioprotective strategies in the management of individuals at risk for T2DM and CVD.

## Figures and Tables

**Table 1:** LV echocardiographic parameters at week 18.

	SED	MIT	HIIT
PWTd (mm)	2.78 ± 0.23	2.08 ± 0.08 **	1.98 ± 0.06 **
PWTs (mm)	3.93 ± 0.18	2.89 ± 0.06 **	3.08 ± 0.15 *
AWTd (mm)	2.39 ± 0.16	2.16 ± 0.15	2.16 ± 0.08
AWTs (mm)	3.96 ± 0.19	3.66 ± 0.22	3.31 ± 0.11 *
EDV/BSA (µL/cm^2^)	0.827 ± 0.048	0.944 ± 0.052	0.866 ± 0.064
ESV/BSA (µL/cm^2^)	0.212 ± 0.020	0.280 ± 0.030	0.296 ± 0.037
Cardiac Index (mL/min/cm^2^)	0.201 ± 0.018	0.201 ± 0.009	0.184 ± 0.017
Radial FS (%)	50 ± 3	41 ± 1 *	40 ± 1 **
EF (%)	75 ± 1	70 ± 3	68 ± 2
HR (bpm)	342 ± 12	331 ± 10	321 ± 14
BSA (cm^2^)	828 ± 11	710 ± 14 ***	715 ± 20 ***

Echocardiographic characteristics at the end of this study of rats that received a Western diet and were kept sedentary (SED, *n* = 7), underwent moderate-intensity training (MIT, *n* = 7) or underwent high-intensity interval training (HIIT, *n* = 8). Data represent means ± SEMs. * denotes *p* < 0.05, ** denotes *p* < 0.01 and *** denotes *p* < 0.001 vs. SED. AWTd: anterior wall thickness in diastole. AWTs: anterior wall thickness in systole. BSA: body surface area. EDV: end-diastolic volume. EF: ejection fraction. ESV: end-systolic volume. FS: fractional shortening. HR: heart rate. PWTd: posterior wall thickness in diastole. PWTs: posterior wall thickness in systole.

**Table 2:** LV hemodynamic parameters at week 18.

	SED	MIT	HIIT
EDP (mmHg)	8.6 ± 0.6	4.8 ± 1.3	6.5 ± 0.8
ESP (mmHg)	109 ± 3	99 ± 2 *	93 ± 2 ***
Tau (s)	0.0163 ± 0.0021	0.0108 ± 0.0007 **	0.0105 ± 0.0004 **
SPTI (mmHg*s)	7.983 ± 0.264	6.734 ± 0.263 *	6.137 ± 0.336 **
Diastolic Duration (s)	0.0848 ± 0.0015	0.0917 ± 0.0044	0.0868 ± 0.0030
Systolic Duration (s)	0.0880 ± 0.0022	0.0799 ± 0.0017 *	0.0851 ± 0.0017

Hemodynamic measurements at the end of this study of rats that received a Western diet and were kept sedentary (SED, *n* = 5), underwent moderate-intensity training (MIT, *n* = 7) or underwent high-intensity interval training (HIIT, *n* = 6). Data represent means ± SEMs. * denotes *p* < 0.05, ** denotes *p* < 0.01 and *** denotes *p* < 0.001 vs. SED. EDP: end-diastolic pressure. ESP: end-systolic pressure. SPTI: systolic pressure–time index. Tau: time constant for isovolumetric relaxation.

## Data Availability

The data presented in this study are available on request from the corresponding authors. The data are not publicly available due to privacy reasons.

## Supplementary Materials

The following supporting information can be downloaded at: https://www.mdpi.com/article/10.3390/nu16030431/s1, Figure S1: Representative LV echocardiographic images and quantification of cardiac injury biomarker protein levels; Figure S2: Perivascular fibrosis in LV tissue; Table S1: Primer sequences used for RT-qPCR.

## Abbreviations

AGEs	Advanced glycation end products
AUC	Area under the curve
AWTd	Anterior wall thickness in diastole
AWTs	Anterior wall thickness in systole
BSA	Body surface area
CD163	Cluster of differentiation 163
CD206	Cluster of differentiation 206
CD68	Cluster of differentiation 68
CD86	Cluster of differentiation 86
CO	Cardiac output
CVD	Cardiovascular disease
dP/dt_max_	Maximum peak time derivative
dP/dt_min_	Minimum peak time derivative
E/A	Ratio of peak mitral flow velocity in early versus late filling
E′	Peak septal mitral annulus velocity in early filling phase
E/E′	Ratio of peak mitral flow velocity versus peak mitral annular velocity
ECM	Extracellular matrix
EDP	End-diastolic pressure
EDV	End-diastolic volume
EF	Ejection fraction
ESP	End-systolic pressure
ESV	End-diastolic volume
FABP	Fatty acid-binding protein
FS	Fractional shortening
GLO1	Glyoxalase 1
HFD	High-fat diet
HFpEF	Heart failure with preserved ejection fraction
HFrEF	Heart failure with reduced ejection fraction
HIIT	High-intensity interval exercise training
HMBS	Hydroxymethylbilane synthase
HOMA-IR	Homeostatic model assessment for insulin resistance
HR	Heart rate
IL-1β	Interleukin 1 beta
i.p.	Intraperitoneally
LOX	Lysyl oxidase
LV	Left ventricular
MGO	Methylglyoxal
MIT	Moderate-intensity exercise training
Myl3	Myosin light chain 3
NADPH	Nicotinamide adenine dinucleotide phosphate
NOX4	Nicotinamide adenine dinucleotide phosphate oxidase 4
OGTT	Oral glucose tolerance test
PWTd	Posterior wall thickness in diastole
PWTs	Posterior wall thickness in systole
RAGE	Receptor for advanced glycation end products
ROS	Reactive oxygen species
RPL13a	Ribosomal protein L13a
RT	Room temperature
RT-qPCR	Real-time quantitative polymerase chain reaction
SED	Sedentary lifestyle
SEM	Standard error of the mean
SOD2	Superoxide dismutase
SPTI	Systolic pressure–time index
T2DM	Type 2 diabetes mellitus
Tau	Time constant for isovolumetric relaxation
TNF-α	Tumor necrosis factor alpha
TnI	Troponin I
WD	Western diet
