# Supplementary material for: Moderate-Intensity and High-Intensity Interval Exercise Training Offer Equal Cardioprotection, with Different Mechanisms, during the Development of Type 2 Diabetes in Rats
Source: Nutrients. 2024 Jan 31;16(3):431. doi: 10.3390/nu16030431 (PMC10856993; doi:10.3390/nu16030431)
Supplement: Supplementary file 1 [file nutrients-16-00431-s001.zip › nutrients-2839146-supplementary.pdf]

nutrients

Supplementary material

1. Supplementary table

Table S1. Primer sequences used for RT-qPCR.

Gene	Forward primer	Reverse primer
**CD163**	ATCACAGCATGGCACAGGT	TCCAGATCATCCGTCTTCG
**CD206**	AAGGTTCCGGTTTGTGGAG	TGCATTGCCCAGTAAGGAG
**CD68**	CACTTGGCTCTCTCATTCCCT	GCTGAGAATGTCCACTGTGCT
**CD86**	GTCAAGACATGTGTAACCTGCACC	ACGAGCTCACTCGGGCTTAT
**GLO1**	GAAGATGACGAGACGCAGAGTTAC	CAGGATCTTGAACGAACGCCAGAC
**HMBS**	TCCTGGCTTTACCATTGGAG	TGAATTCCAGGTGAGGGAAC
**IL-1β**	ACCCAAGCACCTTCTTTTCCTT	TGCAGCTGTCTAATGGGAACAT
**NOX4**	TCATGGATCTTTGCCTGGAGGGTT	AGGTCTGTGGGAAATGAGCTTGGA
**RAGE**	CAGGGTCACAGAAACCGG	ATTCAGCTCTGCACGTTCCT
**RPL13A**	GGATCCCTCCACCCTATGACA	CTGGTACTTCCACCCGACCTC
**SOD2**	AGCTGCACCACAGCAAGCAC	TCCACCACCCTTAGGGCTCA
**TNF-α**	CTTATCTACTCCCAGGTTCTCTTCAA	GAGACTCCTCCCAGGTACATGG

Primer sequences are given in their 5′-3′ orientation. CD163, Cluster of differentiation 163. CD206, Cluster of differentiation 206. CD68, Cluster of differentiation 68. CD86, Cluster of differentiation 86. GLO1, Glyoxalase 1. HMBS, Hydroxymethylbilane synthase. IL-1β, Interleukin 1 beta. NOX4, Nicotinamide adenine dinucleotide phosphate oxidase 4. RAGE, Receptor for advanced glycation end products. RPL13A, Ribosomal protein L13a. SOD2, Superoxide dismutase 2. TNF-α, Tumor necrosis factor alpha.

2. Supplementary figures

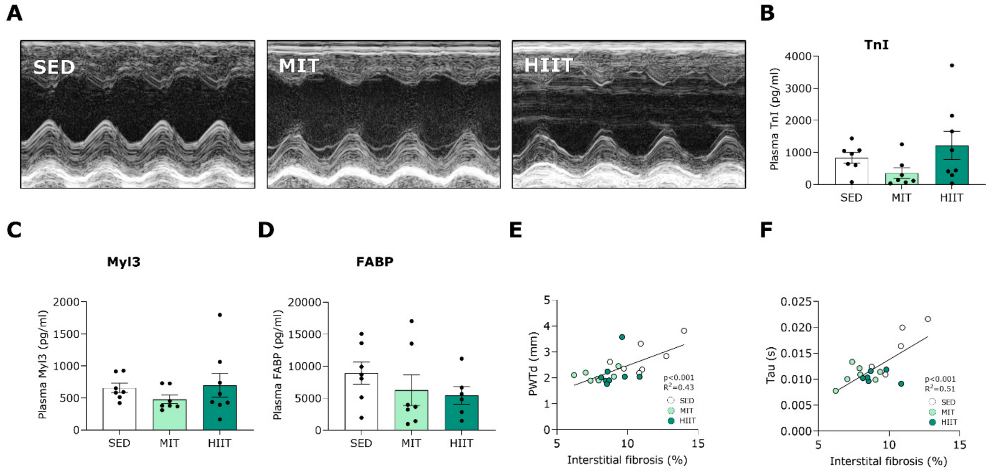

Figure S1. Representative LV echocardiographic images and quantification of cardiac injury biomarker protein levels. (**A**) Representative echocardiographic images obtained in M-mode, parasternal short-axis view. (**B-D**) Quantification of cardiac injury marker protein levels of (**B**) TnI, (**C**) Myl3 in plasma of SED (n=7), MIT (n=7) and HIIT (n=8) and, (**D**) FABP in plasma of SED (n=7), MIT (n=7) and HIIT (n=6). (**E**) Correlation between percentage of LV interstitial fibrosis and PWTd of SED (n=7), MIT (n=7) and HIIT (n=8). (**F**) Correlation between percentage of LV interstitial fibrosis and Tau of SED (n=5), MIT (n=7) and HIIT (n=6). Data represent mean ± SEM. FABP, fatty acid binding protein. LV, left ventricular. Myl3, myosin light chain 3. PWTd, posterior wall thickness in diastole. Tau, time constant for isovolumetric relaxation. TnI, troponin I.

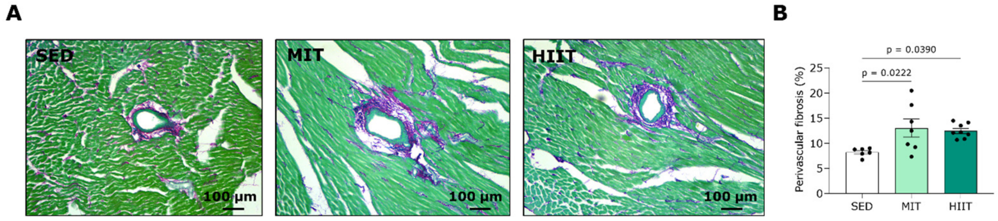

Figure S1. Perivascular fibrosis in LV tissue. (**A**) Representative pictures of LV tissue stained with Sirius Red/Fast Green, zoomed-in on cardiac blood vessels. Fibrotic tissue is stained purple while cardiac cells are stained green. (**B**) Quantification of the percentage of perivascular collagen deposition per surface area in LV tissue from SED (n=7), MIT (n=7) and HIIT (n=8).
